# Effects of digital communication tools on patients, family members and health care professionals in adult ICUs: a mixed-methods systematic review

**DOI:** 10.1186/s13054-025-05826-5

**Published:** 2026-03-03

**Authors:** Eunkyeong Oh, Anett Mueller-Alcazar, Sven Kottysch, Nikolas Groth, Candelaria Irene Mahlke

**Affiliations:** 1 Department of Psychiatry and Psychotherapy, University Medical Center Hamburg-Eppendorf (UKE), Hamburg, Germany; 2 Department of Biological Psychology, Institute of Cognitive and Affective Neurosciences (ICAN), Medical School Hamburg (MSH), Hamburg, Germany; 3 ICF Research Institute, MSH Medical School Hamburg, Hamburg, Germany; 4 MSB Medical School Berlin, Faculty of Medicine, 14197 Berlin, Germany

**Keywords:** Digital communication, Virtual visiting, Intensive care units, Family-centred care, Mixed-methods systematic review, Digital health interventions

## Abstract

**Objective:**

The COVID-19 pandemic accelerated the rapid adoption of digital communication tools in clinical settings. This review aims to identify, synthesize, and critically appraise evidence on digital communication methods or interventions in adult intensive care units (ICUs) intended to promote the psychological and physical well-being of patients and their families, and to explore the associated impacts on healthcare professionals.

**Design:**

Mixed-methods systematic review (MMSR).

**Information sources:**

A systematic search was conducted in MEDLINE, CINAHL, PsycINFO, PSYNDEX, the Cochrane Library, and PROSPERO from 2010 to September 2023 and updated to July 2025. Reference lists and trial registries were screened for additional and ongoing studies.

**Methods:**

Following the JBI convergent integrated approach and PRISMA 2020 guidelines, quantitative data from randomized controlled trials (RCTs) were pooled in random-effects meta-analyses for family satisfaction and patient anxiety. Numerical findings from non-RCTs were qualitized and synthesized narratively. The qualitative data were subjected to thematic synthesis. All results were integrated into a single line of argument.

**Results:**

Fifty-four studies were included, comprising 22 qualitative, 25 quantitative, and 7 mixed-methods designs from 19 countries; 92% were conducted during the COVID-19 pandemic. Over half of the studies examined virtual visiting or video communication (57%, *n* = 31), whereas the others evaluated structured patient-status updates, family support teams, dynamic interaction platforms, or interventions for mechanically ventilated or delirious patients. Methodological quality was moderate to high in 96% of the studies. The meta-analysis of three RCTs demonstrated a moderate to strong improvement in family satisfaction (standardized mean difference = 0.76, 95% CI 0.45–1.06, *p* < .001) with virtual communication compared with usual care. Pooled effects on patient anxiety (mean difference = -2.19, 95% CI -4.62 to 0.23) and depression were nonsignificant, although qualitative findings consistently described perceived reductions in anxiety, loneliness, and emotional distress. Across study types, digital communication enhanced information sharing, supported shared decision-making, and increased family involvement. Key barriers included technical difficulties, privacy concerns, and staff workload, whereas facilitators comprised user-friendly technology, structured preparation, and continuity through a dedicated contact person.

**Conclusions:**

Digital communication in adult ICUs is feasible, acceptable, and beneficial for patients, relatives, and healthcare professionals. Virtual tools improve family satisfaction and complement patient- and family-centred care, but sustainable integration requires clear protocols, staff training, and ethical frameworks beyond pandemic conditions.

**Supplementary Information:**

The online version contains supplementary material available at 10.1186/s13054-025-05826-5.

## Introduction

*Patient- and family-centred care (PFCC)* has been recognized as an important relational concept in critical care since the mid-1980s, when families were first actively integrated into neonatal intensive care units [33]. Since then, increasing evidence has highlighted the benefits of family involvement, particularly for the psychological well-being of patients and relatives, as well as for communication, shared decision-making, and care outcomes in clinical settings more broadly [18, 60, 65]. From the early 2000 s onward, research on patient and family satisfaction and participation in intensive care expanded substantially [36, 68]. Beyond clinical treatment, PFCC emphasizes the psychological and emotional needs of patients and their families by actively involving relatives in information exchange, decision-making, and supportive care processes [44, 72]. However, implementation in practice remains highly heterogeneous and is often constrained by institutional structures and limited staff resources [26, 55, 86].

From the 1990 s to the early 2010 s, PFCC research primarily focused on communication processes and the emotional burden experienced by relatives of *intensive care unit (ICU)* patients [38]. Commonly reported stressors included uncertainty, insufficient information, and limited access to loved ones due to exclusion from care processes [24, 56, 61, 77, 80]. Psychological symptoms such as anxiety and depression are frequently observed among family members of critically ill patients, particularly when structured support is lacking [67, 68]. Moreover, studies have demonstrated that meaningful family participation, e.g., during ICU rounds or daily care activities, builds trust in the healthcare system and reduces relatives’ feelings of helplessness [3, 6, 34].

The COVID-19 pandemic profoundly altered family involvement in ICU care. Widespread restrictions on in-person visitation affected all parties involved, but particularly patients and their families [22, 31]. This unprecedented situation led to a rapid increase in the use of digital communication tools, which partially substituted for physical presence by enabling contact between healthcare professionals, patients, and relatives [37, 73]. Virtual visitation platforms, structured messaging systems, or even asynchronous video updates became important means of maintaining connection and continuity of communication despite isolation policies [53]. Emerging evidence suggests that digital contact can alleviate feelings of helplessness and distress and may enhance the perceived quality of care for patients and families alike [14]. Undeniably, the pandemic accelerated the adoption of telemedicine approaches across healthcare systems worldwide [21].

Early evidence on digital communication for family involvement originated primarily from neonatal and paediatric ICU settings [62, 96]. Interventions such as video messages or structured patient-status updates showed promising results in these contexts [4, 10, 14]. In contrast, empirical evidence from adult ICUs remains comparatively limited and heterogeneous. Although initiatives such as remote ICU rounds, video messaging systems or liaison programmes have been implemented, their evaluation has often been restricted in scope [52, 53, 87]. On the other hand, digital solutions are associated with challenges such as unequal access, inconsistent implementation, and the fundamental limitation that they cannot replace physical presence [37]. Nevertheless, ongoing advances in digital technologies may offer complementary approaches to strengthen communication between patients, ICU staff, and relatives, thereby supporting PFCC beyond neonatal and paediatric settings. This potential is also emphasized in the Family-Centred Care Guidelines of the Society of Critical Care Medicine (SCCM) [40, 42].

Over the past four decades, PFCC has evolved into an internationally recommended standard of care in intensive care medicine. In the context of the COVID-19 pandemic, digital communication has gained a central role in discussions on PFCC, particularly in settings with restricted physical access. However, evidence regarding its effectiveness and impact on patients, family members, and healthcare professionals in adult ICUs remains fragmented.

This systematic review aims to synthesize the available evidence on the effects of digital communication in adult ICU settings. Using a mixed-methods approach, the review integrates qualitative and quantitative findings to provide a comprehensive and practice-oriented understanding of virtual family communication in critical care.

In this review, the term “digital communication” is used as an umbrella concept. The term “virtual communication” refers specifically to synchronous video- or audio-based contact. For consistency, the broader term “telecommunication” is not used.

## Objectives

The goal of this systematic review was to identify, synthesize and appraise evidence of virtual communication methods or interventions in adult intensive care units that emphasize family involvement. These interventions aimed to promote the psychological and physical well-being of patients and their relatives. Additionally, the associated experiences and implications for healthcare professionals were explored.

## Methods

This systematic review was conducted in accordance with the Joanna Briggs Institute (JBI) methodology for mixed-methods systematic reviews. Reporting followed the *Preferred Reporting Items for Systematic Reviews and Meta-Analyses (PRISMA)* 2020 statement. The review process was initiated in 2023 and was retrospectively registered in the PROSPERO database in early 2025 (registration number: CRD42025638475).

### Eligibility criteria

#### Inclusion criteria

Studies were eligible for inclusion if they met the following criteria:

- Population: Adult ICU patients (≥ 18 years), their relatives, and healthcare professionals.
- Intervention: Any digital communication used to support interaction between patients, families, or staff (e.g., video or audio calls, virtual visits, secure messaging, communication platforms, mobile device-based tools).
- Outcomes: Psychological, emotional, informational, or experience-based outcomes for patients, families, or healthcare professionals.
- Study designs: Qualitative, quantitative, and mixed-methods empirical studies.
- Setting: Adult ICU settings.
- Time frame: Publications from 2010 to July 2025.

Studies conducted in critical care settings that examined digital communication interventions in relation to psychological or physical well-being outcomes were included. Quantitative, qualitative, and mixed-methods designs were considered, and eligibility was not restricted to *randomized controlled trials (RCTs)*. The lower time limit of 2010 was chosen to reflect the widespread availability of video-capable mobile devices and secure digital communication platforms that enable structured virtual communication in ICU settings.

Studies were excluded if they were conducted outside of critical care (e.g., nursing homes), focused on pediatric, neonatal, or palliative care settings, or consisted solely of simulation studies or technology development without clinical evaluation. Conference abstracts without full texts and non-empirical publications (e.g., reviews, editorials, commentaries) were also excluded.

### Search strategy

A comprehensive literature search was conducted across multiple electronic databases including MEDLINE (PubMed), CINAHL, PsycINFO, PSYNDEX, PROSPERO and the Cochrane Library for Cochrane Review and the *Cochrane Central Register of Controlled Trials (CENTRAL)*. PROSPERO and CENTRAL were included to ensure the identification of relevant gray literature; however, further searches of clinical trial registries were not conducted. Protocols included after assessment for eligibility without published results were noted but not integrated into the analysis. These are referenced narratively to highlight ongoing research and areas requiring further investigation. The full search strategy is available in the Supplementary Material 1 (Additional File 1).

The search period was restricted from 2010 to 27^th^ of September 2023. Finally, reference lists of identified studies were scanned, and experts of major studies were contacted to identify any potentially relevant published work that may not have been captured during the electronic search. No new data were collected.

### Search update

Given the time elapsed since the initial search and in accordance with current methodological standards for systematic reviews, an updated literature search was conducted on the 3^rd^ of July 2025, using the same databases and strategies, covering the period from the 27^th^ of September 2023 to the 3^rd^ of July 2025.

### Study selection process

**Fig. 1:**
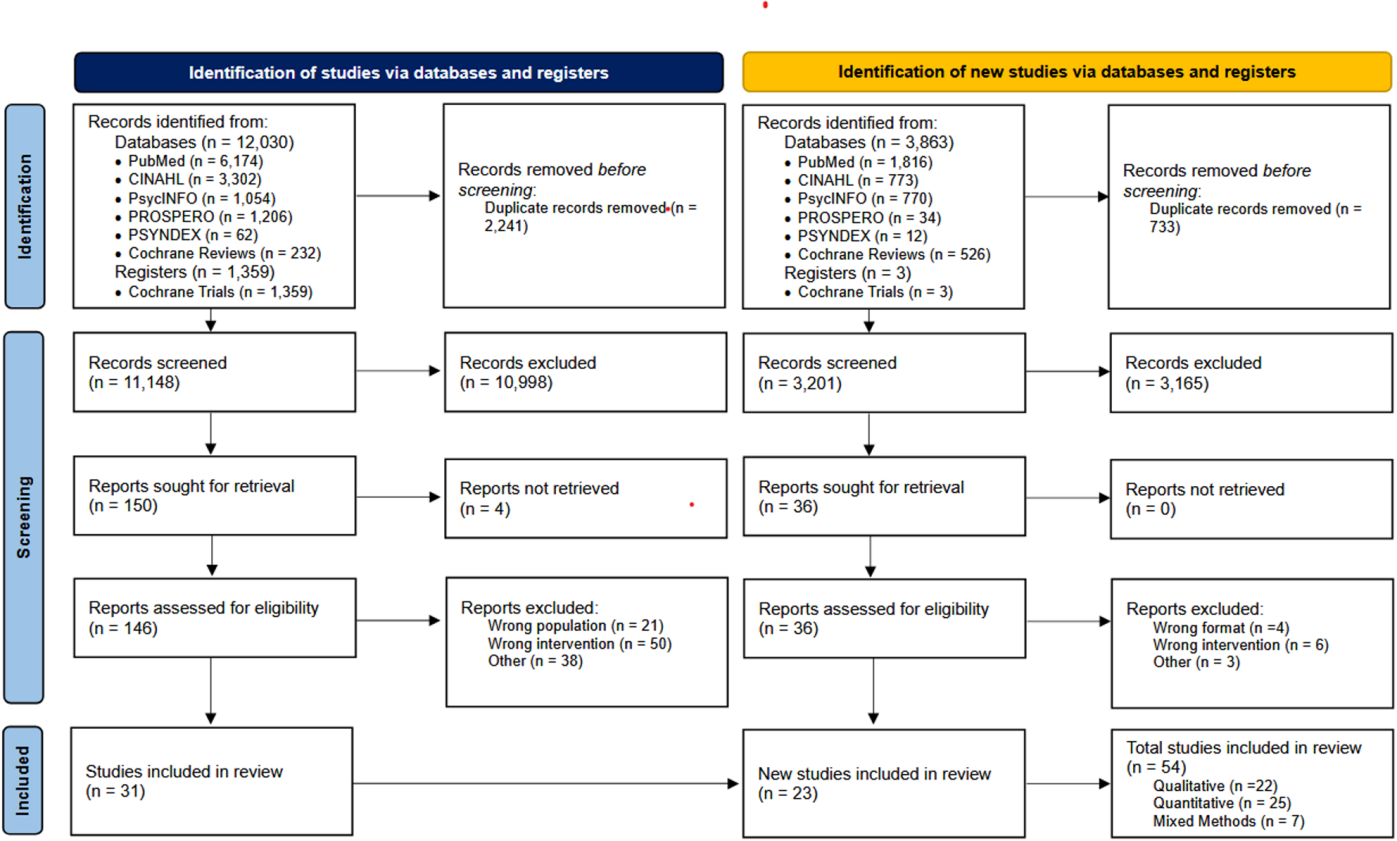
Study selection process across the initial and updated literature searches, including identification, screening, eligibility assessment, and inclusion of studies in the mixed-methods synthesis

Studies searched from each database have been transferred to Rayyan for screening, an application for systematic reviews [64]. After the removal of duplicates, all titles and abstracts were screened in accordance with the predefined criteria by two reviewers independently (November 2023-January 2024 (E.O., A.B.), July 2025-August 2025 (E.O., N.G.)). Every paper judged by either reviewer as relevant was obtained in full text for further screening. Data meeting the inclusion criteria were assessed for relevance. Any discrepancies were resolved by consensus and further consultation by a third party (A.M.A). The study selection process is described with a PRISMA flow diagram in Fig. 1.

### Quality assessment of publications

The methodological quality of the included studies was assessed by two independent reviewers (November 2023-January 2024 (E.O., A.B.), July 2025-August 2025 (E.O., N.G.)) via the *Mixed-Methods Appraisal Tool (MMAT)* [41]. Discrepancies regarding the judgement of quality were resolved through further dialogues. In the initial search phase, no contact with the authors for clarification of the data or a third reviewer for disagreements was needed. In the update search, the authors of one paper were contacted to obtain additional data for the meta-analysis [93]; however, no further information could be retrieved.

### Data extraction

Data extraction was performed independently by two reviewers using the Cochrane Data Extraction and Assessment Form (November 2023-January 2024: E.O., A.B.; July 2025-August 2025: E.O., N.G.). Extracted information included study eligibility, population characteristics, setting, study aims and methods, participant characteristics, intervention details, outcome measures, results, and applicability of findings.

For qualitative studies, the extraction format was adapted to reflect qualitative methodologies. For example, instead of items such as randomization or unit of allocation, information on data collection methods and analytical approaches was extracted, and all relevant thematic content was documented. For mixed-methods studies, both the original quantitative extraction criteria and the adapted qualitative criteria were applied.

### Data synthesis and integration: convergent-integrated approach

This mixed-methods systematic review followed the JBI *convergent integrated approach* to data synthesis. Quantitative and qualitative findings were initially synthesized separately. Quantitative results were subsequently transformed into narrative descriptions (“qualitized”) to enable integration with qualitative data. Qualitative findings were analyzed using thematic synthesis. In the final step, all qualitized quantitative findings and qualitative themes were integrated to develop a comprehensive line-of-argument synthesis across data types.

### Quantitative data synthesis

Quantitative data extracted from eligible studies included numerical outcome data. For randomized controlled trials with comparable outcome measures, meta-analyses were conducted using *standardized mean differences (SMDs)* when different measurement instruments were applied, or *mean differences (MDs)* when the same instruments were used. 95% confidence intervals were calculated. Meta-analyses were performed for three outcomes: family satisfaction, patient anxiety, and patient depression.

All analyses were conducted using random-effects models to account for heterogeneity across study populations, interventions, and measurement contexts. Heterogeneity was assessed using the I² and τ² statistics. Meta-analyses were performed using *Review Manager (RevMan)* version 9.5.1. Quantitative findings from non-randomized studies were also qualitized (i.e., converted into narrative form) and incorporated into the integrated synthesis.

### Qualitative data synthesis

Qualitative data comprised participant experiences and perceptions reported in the included studies. These data were analyzed using the three-stage thematic synthesis approach described by Thomas and Harden: (1) line-by-line coding, (2) development of descriptive themes, and (3) generation of analytical themes [89]. Coding was conducted inductively, and findings were grouped into descriptive categories before being further refined into higher-order analytical themes.

### Mixed-methods data

For mixed-methods studies, both quantitative and qualitative data were extracted. Numerical outcome data and accompanying narrative descriptions were obtained from the quantitative components. Quantitative findings from non-randomized designs were qualitized to enable integration with qualitative themes within the convergent integrated synthesis.

### Data integration

Following separate synthesis of quantitative and qualitative findings, all qualitized data were summarized into overarching themes. These themes were integrated with qualitative narratives to produce a single, simultaneous synthesis that linked intervention effectiveness with participant experiences and implementation contexts.

## Results

The initial electronic database search resulted in 13,389 citations. After removing duplicates, 11,148 citations remained for the screening process. The first screening resulted in 150 studies to retrieve the full-text versions, of which four were not obtainable. According to the inclusion criteria, 111 papers were excluded: for either not examining the appropriate intervention, not having the specified patient population or for other reasons such as insufficient methodological detail and irrelevant study focus, etc. Of the remaining 37 articles, six were trial registers for future studies and therefore, 31 studies were included in the final review.

The update search yielded 3,900 records, of which 733 duplicates were removed, resulting in 3,167 references screened by title and abstract. The full-texts of 36 references were screened by two independent reviewers (E.O., N.G.). Twenty-three additional studies were included. The reasons for exclusion were as follows: four were review articles, six were limited in relevance to the intervention or topic, one focused on a post-ICU intervention, one had an unsuitable format, and one was conducted in a non-ICU setting.

A comprehensive overview of the study selection process is illustrated in the PRISMA flow diagram (Fig. 1). Supplementary Material 2 (Table S1) contains the extracted data from each included study, providing the evidence base for the synthesis of virtual family communication in adult critical care environments.

### Characteristics of the included studies

There are 22 qualitative studies, 25 quantitative and 7 mixed-methods studies among the 54 papers included in this review. Studies were published between 2015 and 2025 and originated from a broad range of countries. The majority of studies were from the United States of America (USA) (*n* = 12) [9, 11, 12, 18, 19, 21, 46, 55, 66, 74, 78, 82] and the United Kingdom (UK) (*n* = 9) [15, 22, 70, 71, 85] *n* = 4) [5, 45, 83, 84], Australia (*n* = 4) [33, 43, 51, 65], Canada (*n* = 3) [7, 13, 48], Switzerland (*n* = 3) [24, 64, 90], and Iran (*n* = 3) [76, 77, 79]. Single studies were published from Italy [78], France [32], Denmark [63], the Netherlands [50], Colombia [27], China [97], Saudi Arabia (SAU) [2], South Korea [93], Ireland [57], Israel [8], Spain [29], and the United Arab Emirates (UAE) [1], as well as one multicountry study from Austria, Germany, Switzerland and Liechtenstein [43]. Among the 54 included studies, most were conducted during the COVID-19 pandemic or post-pandemic (96%, *n* = 52/54) [1, 2, 5, 7–9, 11–13, 15, 16, 18, 21, 22, 24, 26, 28, 30, 33, 40, 42–48, 51, 54, 55, 59, 64–66, 68–71, 73, 74, 76–79, 82–87, 89, 90], with the rest conducted in pre-pandemic contexts [20, 39]. Over half have examined virtual visiting or video communication interventions between patients and family members (57%, *n* = 31/54) [1, 5, 9, 11, 15, 16, 18, 21, 22, 26, 28, 42–44, 46, 48, 59, 68–70, 74, 76–79, 82, 83, 85, 87, 89, 90], followed by broader digital communication tools such as patient status updates from health care professionals with the relatives through telephone calls, messaging (13%, *n* = 7/54) [12, 30, 33, 40, 47, 64, 66], or family support systems (4%, *n* = 2/54) [47, 74] and multimodal systems (4%, *n* = 2/54) [18, 71]. Some studies have investigated digital communication interventions that specifically target mechanically ventilated (MV) patients or delirium management (7%, *n* = 3/54) [8, 42, 43]. Most studies focused solely on family members (31%, *n* = 17/54) [2, 12, 19, 26, 33, 44, 47, 64, 66, 69, 70, 73, 74, 77, 78, 85, 90] or health-care professionals (24%, *n* = 13/54) [7, 11, 13, 15, 16, 18, 28, 54, 55, 59, 68, 83, 87], whereas others combined health-care professionals and family members (14%, *n* = 8/54) [1, 9, 22, 30, 37, 46, 51, 56], and involved both patients and family members (11%, *n* = 6/54) [45, 79, 82, 84, 86, 89] or even included all three stakeholder groups (13%, *n* = 7/54) [21, 24, 40, 42, 43, 48, 71]. Few studies have investigated only patients (7%, *n* = 3/54) [5, 8, 76].

### Methodological quality assessment

Two independent reviewers (E.O., A.S. for the initial search, E.O., N.G. for updated data) assessed the methodological quality of eligible papers via the MMAT. In accordance with MMAT guidance, each study was appraised on the basis of five design-specific criteria. Although the MMAT does not recommend calculating an overall score or ranking, for narrative purposes in this review, studies with one or no criteria rated as “No” or “Can’t tell” are referred to as high-quality, and those with two to three such ratings are referred to as moderate-quality. Although several studies fulfilled most MMAT criteria, important methodological limitations (e.g. representativeness, blinding, nonresponse bias, confounding) were frequent. To avoid overstating study robustness, we refer to these studies as ‘higher quality’ rather than ‘high quality’, emphasizing that these classifications should be interpreted cautiously. The overall methodological quality of the included studies ranged from moderate to high. Among the 54 included studies, 76% (*n* = 41/54) [1, 2, 5, 7, 9, 11–13, 15, 16, 21, 22, 26, 28, 30, 42–48, 55, 59, 64, 65, 68–71, 74, 76, 77, 79, 82–87, 89] were classified as higher-quality, 20% (*n* = 11/54) [18, 19, 24, 33, 37, 40, 51, 54, 66, 73, 78] as moderate-quality, and 4% (*n* = 2/54) [8, 90] as lower-quality.

For all eligible qualitative studies, the type of study design was assessed as adequate. Findings were clearly grounded in the data, providing a coherent analysis and interpretation of the results substantiated by the data. Most studies described data collection methods, with the exception of two studies. For example, Dhala et al. (2020) defines their study as only with the documented work without mentioning the data collection process, and White et al. (2021) limited the data collection to feedback received in a short video call with no predefined questions [23, 92]. de Figueiredo et al. (2024) is categorized as moderate to lower-quality, presenting a brief sketch of the study to shield the quality assessment of its investigation process out of reach [19]. Taken together, the qualitative studies demonstrated high-quality adherence to the MMAT-method, whereas the methodologies reported by Dhala et al. (2020) and White et al. (2021) required a cautious interpretation of their results; their evidential weights compared with those of well-reported qualitative designs are therefore lower [23, 92].

Among the quantitative descriptive studies, all eight were categorized as higher-quality research: all applied appropriate sampling and outcome measurement strategies, enabling the statistical analysis to answer the research question adequately. However, common limitations were found for all the included data except for Rose et al. (2021) and Nelson et al. (2022) [59, 73]. Most studies were conducted with limited representativeness of the sample and had or did not clarify the risk of nonresponse bias for data collection. For example, Levido et al. (2023) and Ramirez et al. (2024) relied on convenience samples or did not report nonresponse characteristics [54, 71], which restricts generalizability relative to stronger examples such as Rose et al. (2021) [73]. Accordingly, their findings contribute insight but should be weighted more cautiously within the synthesis. This limitation should be noted since even being assessed as higher-quality, insufficient representativeness of data in quantitative studies leaves challenges in generalizing its evidence.

In the quantitative non-randomized studies, all the studies used suitable measurements, and the intervention was successfully implemented, with most having complete outcome data. Limited representativeness of the population was observed in less than half of the included studies. One prominent deficit was shown in the control of confounders, almost none of the data addressing this aspect, except for Shirvani et al. (2022) [85], meaning that conclusions drawn from these studies must be treated with caution.

For the quantitative randomized controlled trials, most studies reported appropriate randomization, comparable baseline groups, good adherence to the intervention, and complete outcome data. Nonetheless, the lack of description of the blinding process in many trials cannot be overlooked. Insufficient reporting of blinding procedures in trials such as Suen et al. (2021) and Woo et al. (2024) introduces potential detection or performance bias [88, 93]. Collectively, the eligible quantitative studies in this review are of moderate to higher-quality, with one exception. Given the general description of the study, a precise quality assessment of Bendavid et al. (2025) was impracticable [8]. Therefore, its findings should be interpreted with caution due to methodological limitations.

Finally, the majority of mixed methods studies address the research question well in terms of the use of both quantitative and qualitative methods, integrating the strength of both modalities into coherent outputs. In contrast to most studies showing higher-quality, Zante et al. (2022) barely met any of the quality criteria [98].

In summary, qualitative studies have shown predominantly higher-quality, whereas common weaknesses regarding one question criteria have been observed in quantitative studies. Additionally, most mixed-methods studies were assessed to be of excellent quality. Overall, despite minor weaknesses in individual studies, the methodological quality of the eligible evidence base enables a data synthesis on a sound level. The quality assessment results are presented in detail in Supplementary Material 2 (Table S2).

### Review findings

#### Type of digital communication

Evidence for digital communication in ICU settings has appeared exponentially since the COVID-19 pandemic [7, 11, 12, 47, 51]. Almost half of the included evidence was published in the last two years, between September 2023 and July 2025. A significant increase in the use of new technologies from approximately 60% to 96% has been reported [29], and the range of digital communication in the field has been expanded from merely connecting patients with their relatives to targeting specific symptoms of patients. The most prevalent application of digital communication was audio-visual visiting of relatives with their loved ones. Many pre-existing video conferencing programs, such as Skype, Zoom, and new tools, such as WeChat, Life Lines, and MyVisit have been developed to enable virtual communication [2, 7, 19, 20, 22, 23, 25, 27, 47, 54, 57, 59, 70, 73–75, 81, 91, 93, 95, 97, 98]. Families can send content such as photos, music or videos to patients or be at the patients’ bedside virtually [76, 78]. Some virtual visiting (VVs) are purposefully organized as structured virtual patient visits (sVPVs) [16, 48]. These digital visits also functioned as complements to ICU wards, extending the reach of families as virtual family centred rounds [19, 50, 71]. Another type of the digital communication had the purpose of providing a unidirectional patient status update for the relatives. Doctors and nurses provided patients’ clinical state via audio-visual calls or messages [32, 63, 69, 82, 85, 92]. Digital texting platforms or real-time SMS updates at specific landmark events have been implemented [12, 35]. In addition, different family support systems serve patients’ families via digital tools, dynamic interaction platforms or family support teams (FSTs) via phone calls [5, 37, 47, 51, 82]. Furthermore, communication aids for MV-patients or delirium prevention and intervention were invented [8, 46, 84]. Finally, digital communications involves specialist consultation or even palliative care, facilitating the connection between patients and relatives to say goodbye at the end of life [11, 13, 23, 92].

#### Impact on families

Many positive effects reported by families occurred in the context of pandemic-related visiting restrictions, where virtual visiting was often the only available option. Hence, digital communication mainly serves as the means for meetings with patients and updates for their relatives [54]. The evidence shows the prevailing benefits of VV in participating families [22, 50, 54]. First, virtual communication provides emotional support, and families feel reassured, relaxed and comfortable [9, 23, 35, 48, 50, 69, 74, 92]. Relatives experienced positive sentiments such as happiness, joy, gratitude, and relief [79]. Additionally, many studies address the positive effect of digital communication on families by reducing distress/anxiety and increasing satisfaction [20, 25, 27, 69, 74, 82, 91–93, 97]. Although no significant effect could be proven, one study suggested a relationship between digital intervention and lower levels of anxiety, and PTSD-related symptoms in ICU family care givers post-ICU discharge [84]. VV also promote family involvement, allowing them to participate in discussions and decision making [92, 95]. It supports families who are geographically distant to connect with patients and helps restore family units [51, 76, 95]. A few studies reported family distress through contact, such as overwhelming emotions in difficult situations, feeling burdened as primary family contact, or not being sufficient as a substitute for physical presence [22, 47, 50]. However, evidence of adverse impacts on family members is scarce and fewer deterrent effects are described than among HCPs, with the least common emotions being anger or fear [20, 22, 70, 74]. Overall, digital communication was observed to be highly beneficial for families fulfilling their psychosocial needs to connect with their loved ones [15, 19, 23, 51, 54]. It is welcomed as an innovative support tool and is recommended for continued use by nearly all family participants [48, 54].

#### Impact on patients

Similarly, digital communication has been shown to meet the psychosocial needs of patients, connecting them with their family members [15, 19, 23, 51]. Virtual visits serve as a window into patients’ home and normal everyday lives, increasing their level of satisfaction [19, 25, 63, 91, 97]. The virtual presence of family members emotionally supports patients, mitigate their loneliness and alleviates their anxiety [5, 19, 25, 81, 85, 93]. Unlike patient anxiety, the literature on patient depression is mixed: some studies demonstrate a meaningful reduction, whereas others detect no effect, including on PTSD symptoms [78, 81, 93, 97, 98]. Interestingly, several virtual interventions for delirium prevention and intervention started to emerge within the last few years [8, 45, 46, 97]. Although none of the included studies demonstrated a clear effect on delirium, reorientation interventions with family voices were assessed as acceptable, feasible and emotionally supportive, and remain promising for exploration [45, 46]. Additionally, one study investigated the effects of virtual communication on patients’ vital signs and revealed temporary significant changes in heart rate (HR), respiratory rate (RR), Glasgow Coma Scale (GCS) score and pulse rate (PR) [91]. Overall, digital communication promoted patient centred care (PCC) by assisting patient recovery psychologically and physically. Patients described seeing or hearing family members via virtual contact as motivating; it provided reassurance, emotional encouragement, and a sense of connection during isolation, supporting psychological activation and strengthening patients’ willingness to engage in recovery [45, 48, 73].

#### Impact on HCPs

The introduction of digital communication during the pandemic has shown a different trajectory of impact on physicians and nurses on site. For HCPs, the main purpose of VV is audio-visual calls with patients’ families [54]. A noticeable ambivalence in the HCPs’ experiences was recognized. On the one hand, studies report a decreased workload, an absence of task interruptions and reduced referrals to hospitals, saving physicians and nurses time [12, 32, 48, 63, 69, 81, 85, 90]. Others mention an increased burden on staff through additional phone calls with family and that nurses experience physical and psychological stress [11, 13, 16, 22]. It also impedes the workflow if the inpatient telehealth infrastructure is not well established, challenging nurses in setting up calls or documenting electronic medical records [46, 76]. Nonetheless, VVs fulfill the moral instincts of HCPs and could be recommended to be used continuously in the future [15, 39].

#### Effectiveness

In addition to the effects on patients’ and family members’ satisfaction and anxiety, digital communication has been proven to be an effective medium for information sharing and shared decision-making [49, 88]. It was favourably deployed for low-stake informational communication, delivering faster communication efficiently, and less for high-stake content, such as prognosis discussions or breaking bad news [13, 49]. The communicated medical messages were understandable and valued by family members [32, 71]. Digital tools meeting the information needs of relatives support them gaining insight into patient treatment and thus in rapport building [11, 35, 47, 49, 51, 63, 69]. Another major strength of digital communication in ICU-settings is cost- and time-effectiveness [2, 81, 82]. The system is easy to facilitate, safe and thus saves time and cost for all participating parties.

#### Feasibility, acceptance and usability

Video calls and SMS-update services were evaluated to be feasible and highly acceptable, especially by family members but also by ICU providers [69, 78]. Although challenges related to acceptance have appeared, virtual communication is seen as valuable in patient care by ICU providers and as applicable for all ICU patients, who are conscious or sedated [23, 48, 59]. Digital communication media are evaluated as having an overall high usability, with users being willing to use it again in the future [2, 7, 57].

#### Ethical and legal considerations

Ethical concerns are paralleled by the usability of digital communication in critical care, which has been repeatedly mentioned in several studies [12, 15, 19, 23, 25, 49, 63]. Issues range from insecure transmission of patient images and inconvenience caused by security features to the management of emotional distress [12, 15]. Dilemmas about preserving privacy and providing equitable access to VV technologies at the same time were observed [12, 13, 51, 73].

#### Challenges and barriers

The most frequent barriers were technical difficulties such as device challenges, connectivity problems or user skills [13, 19, 43, 50, 51, 73, 75, 79]. Older patients often had limited experience in technology, and some users expressed criticism of the platform structure, e.g., for lacking images, a home button or for some links to be too hidden [13, 39, 43]. Device access is not always guaranteed for everyone, and unreliable audio-visual qualities or incorrect camera position impedes the flow of communication [73, 75, 95]. Another main obstacle was related to organizational issues. Families complain about having no consistent contact person and thus not being able to experience continuity in service [50]. Unstructured communication processes, a lack of staff training, the absence of standard operating procedures, and highly restrictive visiting policies often lead to unmet expectations and hinder rapport-building among patients, families and staff [50, 75]. Interpersonal challenges are caused mainly by insufficient staff resources, which impose a heavy burden on service providers [2, 19, 43, 75]. In addition, a minority of relatives feel overwhelmed with medical language during virtual family rounds, complicating communication [6].

#### Facilitators

An easy-to-use technology that is accessible and flexible could make inclusive digital communication easier [51, 75]. Structured preparation of relatives and patients using setup resources, schedule coordination and a point-person family support system could strengthen the continuity of service [1, 12, 13, 51, 75]. The presence of ICU members and staff compassion were also mentioned as good facilitators [12, 75].

#### Suggestions and future directions

Several areas of improvement emerged including technology, communication structures, guidelines and equity policy. First, on-demand access, user-friendly interfaces, improved availability of devices and clearer protocols for virtual visits could support the handling of technological issues [12, 15, 39, 73, 75, 79]. Second, communication structures may be ameliorated through dedicated contact persons on site in regular timeframes, structured daily updates of patient status and better integration with the care team [1, 7, 43, 47, 50, 79]. Third, both clinical and ethical guidance for digital communication can prevent staff from being isolated in decision-making and appropriate staff training accordingly will increase the efficiency of service deployment [15, 43, 73]. Such guidelines may also help participants with emotional self-preparation to reduce emotional strain during audio-visual contact [75]. Furthermore, visiting policies could be revised to encourage regular family involvement both in person and virtually, ensuring inclusivity for all relatives through program expansion [1, 76]. Finally, aftercare aspects emerged as an additional theme, highlighting the difficulties families faced when daily calls stopped abruptly after ICU discharge and the need for clearer communication on prognosis and treatment plans beyond the ICU stay [1, 50].

### Meta-analysis

#### Family satisfaction

A meta-analysis was conducted on three randomized controlled trials reporting the effect of digital interventions on family members’ satisfaction [69, 93, 97]. The mean values of the outcomes at post-intervention were retrieved for analysis. Since these studies applied different outcome instruments, standardized mean differences (SMDs) and the random-effects model were used.

**Fig. 2:**
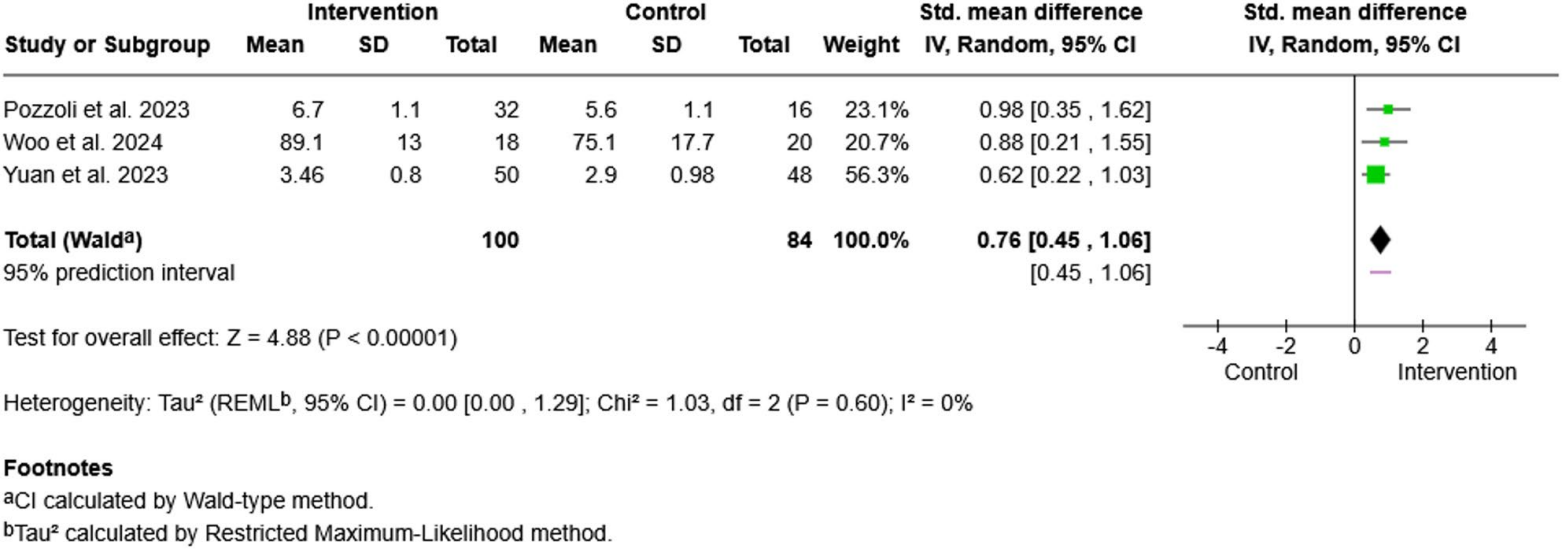
Forest plot of family satisfaction. Forest plot for the effect of digital communication interventions on family satisfaction. Three randomized controlled trials were included. Standardized mean differences (SMDs) were calculated via a random-effects model. Positive SMDs indicate greater satisfaction in the intervention group than in the with usual care group. Heterogeneity was assessed with the I² statistic as very low (I² = 0%)

Data from the study by Yuan et al. (2023) were transformed, since family satisfaction was reported in four ordinal categories (“very satisfied”, “satisfied”, “neutral”, “dissatisfied/very dissatisfied”) [97]. For inclusion in the meta-analysis, numerical values were assigned from 1 (lowest satisfaction) to 4 (highest satisfaction) for each category. Using weighted calculations, the means and standard deviations were estimated on the basis of the frequency distributions across categories (Fig. 2).

The meta-analysis revealed a statistically significant overall effect of digital communication interventions on family satisfaction (SMD = 0.76, 95% CI [0.45, 1.06], Z = 4.88, *p* <.00001). Statistical heterogeneity among the studies was very low (Chi² = 1.03, *df* = 2, *p* =.60; I² = 0%), indicating high consistency across studies. The pooled effect was largely influenced by Yuan et al. (2023), with more than half of the total weight [97]. This analysis suggests a moderate to strong effect of digital communication tools on family satisfaction with ICU communication compared with usual care.

#### Patient anxiety

**Fig. 3:**
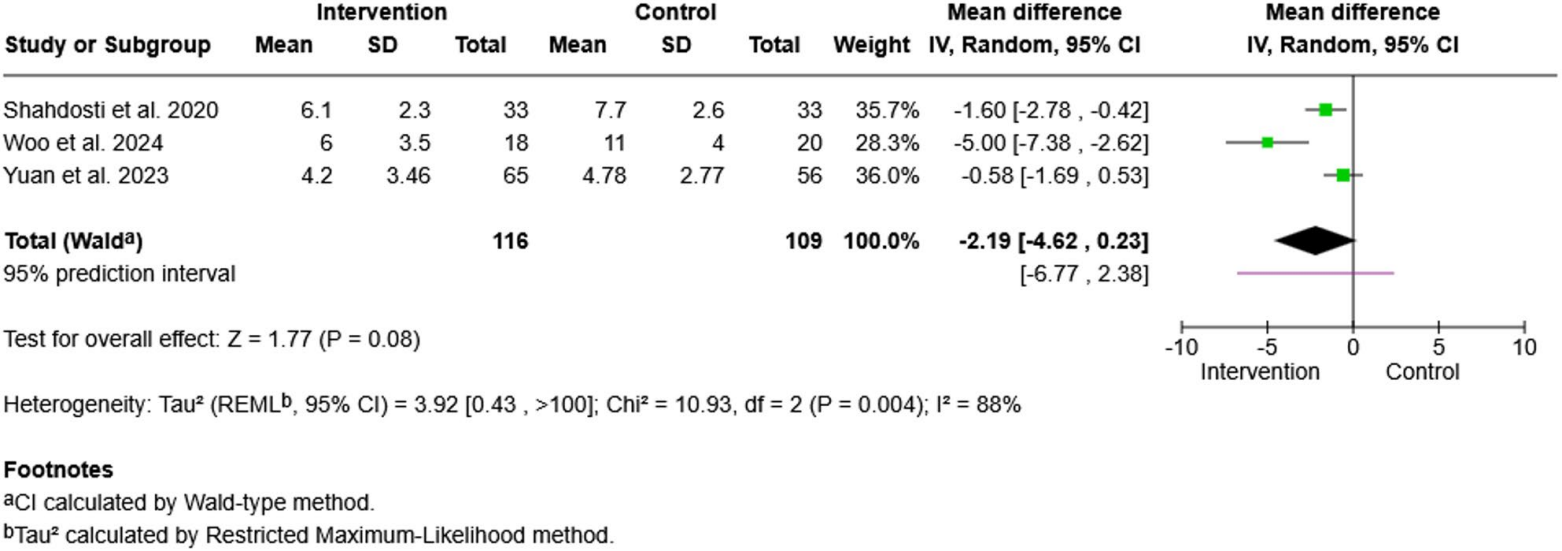
Forest plot of patient anxiety. Forest plot for the effect of digital communication interventions on patient anxiety. All three included randomized controlled trials measured anxiety via the Hospital Anxiety and Depression Scale - Anxiety subscale (HADS-A). Mean differences (MDs) were calculated with a random-effects model. Negative MDs indicate lower anxiety scores in the intervention group. Heterogeneity quantified using the I² statistic was substantial. (I² = 88%)

A second meta-analysis was conducted to evaluate the effects of digital communication interventions on patient anxiety. The mean values of the outcomes at post-intervention were retrieved for analysis. All three randomized controlled trials assessed post-intervention anxiety scores via the Hospital Anxiety and Depression Scale - Anxiety (HADS-A) subscale (HADS-A; range 0–21, with higher scores indicating greater anxiety), allowing for direct comparisons via mean differences (MDs) using a random-effects model [81, 93, 97] (Fig. 3).

The pooled result demonstrated a nonsignificant overall effect in favour of the intervention (MD = −2.19, 95% CI [−4.62, 0.23], Z = 1.77, *p* =.08). There was considerable heterogeneity (Chi² = 10.93, *df* = 2, *p* =.004; I² = 88%), indicating substantial inconsistency across studies. Whereas Shahdosti et al. and Woo et al. reported reductions in patient anxiety, the between-group differences reported by Yuan *et al*. are small [81, 93, 97]. Nonetheless, the number of eligible trials (*n* = 3) did not permit meaningful subgroup or sensitivity analyses. Since all three studies were assessed with high quality according to the MMAT, the heterogeneity may be attributed to the variability in the population or intervention.

## Discussion

### Findings and future directions

This mixed-methods systematic review synthesized evidence from 54 studies and demonstrated that digital communication has become an increasingly relevant component of PFCC in adult ICUs [94]. According to the included studies, digital communication emerged as a highly valuable tool for patients, relatives, and staff on site, even for those whose trajectories varied from each other. These findings align with those of previous reviews that identified digital communication as a rapid response strategy to maintain FCC under restricted visitation policies during the COVID-19 pandemic [30, 66].

Consistent themes across the evidence include emotional support for families, perceived psychosocial benefits for patients, and ambivalent yet substantive experiences among healthcare professionals [58, 83].

Virtual visits provided family members with emotional support, increased their satisfaction and encouraged their involvement in the process of patient care. A meta-analysis confirmed the impact of digital communication on family satisfaction to be moderate-to-strong, underscoring the psychosocial benefits of digital interventions. This aligns with prior reviews reporting improved family satisfaction despite methodological heterogeneity [17]. For patients, digital communication also supported their psychosocial well-being, mitigated anxiety and loneliness, and was perceived as supportive of recovery. The effects on patients’ depression and PTSD symptoms remain inconsistent; however, the majority of digital interventions had no effect. Comparable heterogeneity in psychological outcomes has been repeatedly noted [17, 83]. Considering the characteristic of ‘depression’ as a psychometric outcome, which usually manifests over a longer period from three to six months, these inconsistencies might have resulted from inappropriate time measurements, as none of the studies were conducted over such extended periods.

High heterogeneity was also described in meta-analytic findings on patient anxiety, indicating a discrepancy between qualitative data - patients subjectively reporting reduced anxiety - and quantitative RCT data. Previous reviews similarly reported measurement mismatch and insufficiently standardized intervention delivery [17, 66]. This may reflect measurement timing (often within the first 48–72 h), limited sensitivity of anxiety scales to relational and contextual factors (e.g. emotional reassurance may be mirrored in narratives but not captured by psychometric scales), or substantial variation in intervention delivery. These discrepancies could be investigated in further research with representative sampling and robust study designs.

Importantly, the interpretation of these findings must consider the pandemic context. During the COVID-19 pandemic, virtual communication has acted largely as a substitutional strategy for suspended family presence [17, 30, 66]. Thus, improvements in family satisfaction, anxiety, or psychological well-being were frequently related to highly restrictive visiting policies rather than to standard, pre-pandemic in-person visits.

Beyond the pandemic context, the evidence synthesized in this review indicates that digital communication in adult ICUs extends beyond a substitutional function. Several included studies were conducted in post-pandemic contexts or examined digital communication tools that were not primarily designed as visitation substitutes. These interventions included structured communication platforms, asynchronous messaging systems, and patient-centred digital tools supporting orientation, reassurance, and symptom management, including delirium-related interventions. In these contexts, digital communication was described as facilitating emotional reassurance, continuity of information, and patient engagement, particularly when physical presence was limited. Therefore, despite reported limitations, including emotional burden and reduced contact quality compared with in-person interaction, the overall evidence suggests that digital communication may serve as a complementary component of patient- and family-centred care beyond crisis conditions, rather than being confined to emergency visitation policies.

Interestingly, health care providers expressed prominent ambivalence regarding the use of virtual communication, perceiving it as both beneficial and burdensome. Previous evidence has shown that digital communication improves workflow efficiency but simultaneously increases workload and emotional strain during surges [58, 66]. Digital communication facilitated communication and improved workflow efficiency by reducing referrals, yet additional demands impeded care time, contributing to emotional strain in organizational chaos. These findings highlight the necessity of establishing of adequate infrastructure and implementation guidelines, ensuring the required resources and training for successful implementation in the future.

Across the data, recurrent facilitators and barriers to practical implementation were categorized into the following themes. Facilitators included accessible and user-friendly technology, continuity ensured by a dedicated contact person and structured preparation of participants for virtual communication. Barriers primarily stem from technical or organizational factors, such as user skills, poor connectivity, lack of training, and limited staff resources. These implementation barriers reflect organization-dependent variability already emphasized in prior reviews [30, 58, 66]. According to one study, when supported by structured roles and workflows, digital tools can alleviate the communication burden although capacity constraints (e.g., limited care provider resources) may hinder implementation [58]. These findings underscore the crucial role of a well-structured and collaborative organizational framework in the successful implementation of digital interventions in ICUs. In practical terms, this evidence highlights the need for structured implementation plans, dedicated staff roles, and stable technical infrastructure as prerequisites for sustainable adoption.

Finally, in line with the ethical and accessibility constraints reported from pre- and post-pandemic syntheses, findings emphasize significant ethical dilemmas in digital ICU communication [17, 66]. Concerns regarding patient privacy, equity of access, and data security remain major challenges necessitating that future strategies be developed within validated ethical frameworks and institutional policies. Taken together, these findings highlight the growing policy relevance of digital communication in critical care, underscoring the need for institutional standards that formally set up digital tools within routine ICU practice. Such policies are essential to enable a more inclusive, secure and ethical sound integration of digital communication in ICU care.

According to the framework from Eysenbach, eHealth should primarily aim to increase efficiency, enable evidence-based practice, enhance communication and promote equitable access to healthcare across the population [28]. In light of the accelerating digitalization of medicine globally and on the basis of converging evidence, this review synthesis suggests that digital communication should be considered not solely as a crisis-driven solution but also as a sustainable component of PFCC [17, 66] within ICU care systems. Rather than replacing in-person family presence, digital communication should be construed as a complementary tool that supports interpersonal connection when physical presence is limited or impossible.

Future research should examine a broader spectrum of digital interventions beyond virtual visits, including interactive information platforms for families, chat-based communication systems and self-directed patient communication tools that were co-designed and evaluated in collaboration with end users. Furthermore, future studies should use randomized controlled and longitudinal designs to assess long-term outcomes, including Post-Intensive Care Syndrome (PICS) and Post-Intensive Care Syndrome-Family (PICS-F) outcomes, as well as psychosocial and functional aspects of care and recovery for both patients and their relatives. Intervention development should be guided by the Family-Centred Care guidelines of the SCCM [42], with emphasis on sustainable, user-oriented integration into daily ICU practice. 

### Strengths and limitations

In this mixed-methods systematic review, we synthesized 54 studies, providing the most comprehensive overview of the impact of digital communication on patients’ and families’ physical and psychological recovery in adult ICUs to date. While previous reviews focused primarily on virtual visiting during the acute phase of the COVID-19 pandemic [17, 30, 66, 94], the findings of this review demonstrate that digital communication in the ICU has evolved into a broader field of practice. By integrating qualitative, quantitative, and mixed-methods studies, this review offers a comprehensive scope of evidence with an extended temporal horizon that includes post-pandemic research. Moreover, the inclusion of exploratory meta-analyses strengthens the precision of quantitative outcomes, particularly with respect to family satisfaction and patient anxiety. This broader analytical perspective advances earlier research syntheses that were constrained by limited scope or lower methodological rigor [58, 83].

However, certain limitations should be acknowledged. First, the restriction to studies published from 2010 onwards may have excluded earlier work involving telephone-based communication. Although this review focused on modern audiovisual digital communication, this cut-off remains a potential source of selection bias. Second, although the overall quality of the included studies was moderate to high, several studies presented methodological shortcomings, including small sample sizes and limited representativeness. This reflects ongoing concerns highlighted in prior reviews about insufficient representativeness and the predominance of convenience samples in digital ICU communication research [58, 83]. Third, the absence of blinding, presence of nonresponse bias, and uncontrolled confounding factors in some studies may have introduced potential sources of bias. While the quality assessment identified variability in methodological rigor and reporting transparency across individual studies (e.g. insufficient detail on sampling, data collection or confounder management), differentiated weighting of evidence according to study quality was not applied. Instead, these observations are acknowledged as interpretive boundaries. Accordingly, the pooled quantitative findings should be interpreted with caution. Fourth, this review included studies with a wide range of heterogeneity in interventions and outcome measures, limiting the comparability of evidence across studies, which was similarly identified as a structural limitation in previous evidence syntheses [83]. Fifth, some methodological limitations should be concerned in the meta-analysis. The meta-analysis was conducted with a small number of studies, which restricts the statistical power of its conclusions. Additionally, the heterogeneity of the studies in the patient-anxiety meta-analysis was substantial, indicating the need for further detailed explorations (e.g., sensitivity analysis, subgroup analysis). Given the very limited number of randomized trials, this was not feasible. Moreover, in one study, ordinal categorical data were transformed into continuous data for the purpose of meta-analysis. This procedure, which assumes equal intervals between scale points, may affect the precision of the synthesized effect sizes. We aimed for consistency in these transformations, but the results should be interpreted with caution regarding this statistical assumption. Finally, the predominance of studies from high-income countries limits the generalisability of our findings to resource-constrained settings, where digital infrastructure and staffing capacities may differ substantially.

Future research should prioritize the evaluation of digital interventions using rigorous study designs that assess long-term quantitative outcomes, including depression, delirium, and psychosocial health. As recommended in previous research, additional methodological standardization is needed to improve the comparability of outcomes across study settings [83]. Methodologically comparable trials are needed to reduce heterogeneity and strengthen the evidence base on the clinical and psychosocial impact of digital communication in critical care.

## Conclusions

The evidence suggests that digital communication, which is perceived as acceptable, usable, and beneficial at the trilateral level, facilitates PFCC in the ICU. Digital communication can be a powerful tool to foster family involvement, enhance patient well-being, and support healthcare professionals in critical care beyond the context of the pandemic. Long-term effects and post-ICU aftercare challenges following discharge should be investigated in non-crisis settings to evaluate digital media as a sustainable complementary model in critical care.

## Supplementary Information

Supplementary Material 1: Search Strategy

Supplementary Material 2: Table S1, Table S2

## Data Availability

All the data generated or analysed during this study are included in this published article and its supplementary information files.

## Declarations

### Use of artificial intelligence tools

The authors acknowledge the use of ChatGPT (OpenAI) to support language editing during the revision process. The authors take full responsibility for the content of this manuscript.
