# Supplementary material for: Effects of digital communication tools on patients, family members and health care professionals in adult ICUs: a mixed-methods systematic review
Source: Crit Care. 2026 Mar 3;30:122. doi: 10.1186/s13054-025-05826-5 (PMC13007353; doi:10.1186/s13054-025-05826-5)
Supplement: Supplementary file 1 — Supplementary Material 1: Search Strategy [file 13054_2025_5826_MOESM1_ESM.docx]

**Supplementary Material**

**Additional File 1: Search Strategy**

(("hospitali*"[Title/Abstract] AND ("inpatients"[MeSH Terms] OR "inpatient*"[Title/Abstract])) OR "loneliness"[MeSH Terms] OR "social isolation"[MeSH Terms] OR "isolation"[Title/Abstract] OR "pandemics"[MeSH Terms] OR "pandemic*"[Title/Abstract] OR "intensive care units"[MeSH Terms] OR "intensive care units"[Title/Abstract] OR "icu"[Title/Abstract] OR "critical care"[MeSH Terms] OR "critical care"[Title/Abstract] OR "intensive care"[Title/Abstract] OR "critical illness"[MeSH Terms] OR "critical illness"[Title/Abstract] OR "ITU"[Title/Abstract] OR "CCU"[Title/Abstract])

AND ("involv*"[Title/Abstract] OR "telehealth"[Title/Abstract] OR "telemedicine"[MeSH Terms] OR "telemedicine"[Title/Abstract] OR "telecommunications"[MeSH Terms] OR (("virtual*"[Title/Abstract] OR "remote*"[Title/Abstract] OR "tele*"[Title/Abstract] OR "video*"[Title/Abstract] OR "video call*"[Title/Abstract] OR "Videoconferencing"[MeSH Terms] OR "phone*"[Title/Abstract] OR "tablets"[Title/Abstract] OR "tablet"[Title/Abstract] OR "devices"[Title/Abstract] OR "digital*"[Title/Abstract] OR "Communication system"[Title/Abstract]) AND ("communication"[MeSH Terms] OR "communication*"[Title/Abstract] OR "visit*"[Title/Abstract])))

AND ("professional family relations"[MeSH Terms] OR "family"[MeSH Terms] OR "family"[Title/Abstract] OR "families"[Title/Abstract] OR "relatives"[Title/Abstract])

NOT ("Intensive Care Units, Pediatric"[MeSH Terms] OR "pediatric Intensive care"[Title/Abstract] OR "Intensive Care Units, Neonatal"[MeSH Terms] OR "newborn intensive care"[Title/Abstract] OR "neonatal intensive care"[Title/Abstract])
