# Supplementary material for: Effects of digital communication tools on patients, family members and health care professionals in adult ICUs: a mixed-methods systematic review
Source: Crit Care. 2026 Mar 3;30:122. doi: 10.1186/s13054-025-05826-5 (PMC13007353; doi:10.1186/s13054-025-05826-5)
Supplement: Supplementary file 2 — Supplementary Material 2: Table S1, Table S2 [file 13054_2025_5826_MOESM2_ESM.docx]

**Table S1**. Characteristics of included studies.

| **Author, year (country)** | | **Type of Study** | **Setting** | | | | **Participants** | | | **Type of intervention** | | | **Outcome** | | | **Results** | | | |
| --- | --- | --- | --- | --- | --- | --- | --- | --- | --- | --- | --- | --- | --- | --- | --- | --- | --- | --- | --- |
| ***Qualitative studies*** | |  |  | | | |  | | |  | | |  | | |  | | | |
| Dhala et al., 2020  (USA) | | Case Report | ICU | | | | Patients, families, clinicians | | | Virtual intensive care unit (vICU) via links sent to smartphones (Virtual family visitation, Palliative care delivery, Specialist consultation) | | | Feasibility, acceptance, process-related insights regarding virtual ICU communication during COVID-19 | | | - advantageous mechanism delivering critical care expertise - connecting patients-family (emotional support, improved patient care, satisfaction) - challenges related to security, acceptance | | | |
| Dhahri et al., 2021  (UK) | | Qualitative Study | COVID-19 wards | | | | Relatives (*n* = 108)  Staff (*n* = 30) | | | Video-calls via software "Attend Anywhere" via iPad | | | - Number of video calls - Impact on staff and family members | | | - more positive comments from relatives (appreciation)  - more negative comments in the staff cohort (emotional strain)  - overwhelming emotions, difficult situations | | | |
| Kennedy et al., 2021  (USA) | | Qualitative Study | ICU | | | | Family (*n* = 21)  Clinicians (*n* = 14) | | | Telecommunication (Video-, phone calls) | | | - Effectiveness of phone/video conversations - Concerns about privacy - Comfort with technology | | | - favorably for low-stakes communications (informational updates)  - less favorably for high-stakes discussions  - readily available tablet devices with video capability ameliorate clinician concerns about privacy  - functioned well for information sharing  - concerns about non-informational aspects | | | |
| Sasangohar et al., 2021  (USA) | | Qualitative Study | ICU | | | | Family (*n* = 59) | | | Virtual visiting on the vICU | | | - Feelings - Barriers - Areas for improvement | | | - sentiments / overall experiences: overwhelmingly positive (happiness, joy, gratitude, relief)  - Barriers, concerns, challenges:  (1) keeping in touch with their patients  (2) inquiring health status / care processes administered  (3) anticipating loved one’s future course and timeline  (4) questions to providers regarding care, processes or plans, or add any other concerns  (5) Inability to communicate due to patient status  (6) Technical difficulties  (7) Lack of touch and physical presence  - potential improvements:  (1) On-demand access  (2) Improved communication with care team | | | |
| **Author, year (country)** | | **Type of Study** | | | **Setting** | **Participants** | | | **Type of intervention** | | | **Outcome** | | | **Results** | | |  |  |
|  | |  | | |  |  | | |  | | |  | | | (3) Improved process | | |  |  |
| Klop et al., 2021  (Netherlands) | | Qualitative Study | | | ICU | Family (*n* = 21) | | | Family Support Teams (SFTs) Service: non-ICU staff telephone call with relatives | | | - Well-being of the relative and current situation - Experience with support of SFTs   - Expectations of FST | | | 1) important pillars of the FSTs: emotional support, information  2) combining daily calls from FST with support from the ICU ward: complementary  3) being a primary family contact: valued but also burden  4) role of aftercare  * Certainty: families appreciated small timeframes + fixed contact person  * Prolonged aftercare: difficulties coping with the sudden stop of daily calls after discharge or death  * Video Calling: overall positive + barriers (technical difficulties, technological literacy (both by relatives and staff), difficult and unstructured communication, difficulty in building rapport, lack of continuity (no consistent contact person) | | |  |  |
| White et al., 2021  (UK) | | Qualitative Study | | | COVID-19 wards | Family (*n* = 22) | | | 20Min video call updates from a doctor, end of life video visiting with patients for relatives | | | Feedback of relatives using video calls with doctors (+ patients in end-of-life care) | | | - increased satisfaction around the communication - improved understanding - felt reassured - enabled relatives to be involved in the discussion, provide emotional support - end-of-life care: enabled families to say goodbye to their loved ones and to come to terms with their deterioration  - Challenge: lack of non-verbal communication | | |  |  |
| Rose et al., 2022  (UK) | | Qualitative Study | | | ICU | Family members of COVID-19 patients (*n* = 41) | | | Virtual visiting (Life Lines): one-way initiation of bi-directional calling | | | - Facilitators/barriers of virtual visiting between ICU patients and family during visiting restriction | | | - VV facilitators: preparation of family, negotiating preferred time, easy-to-use technology, ICU member presence, inclusivity, accessibility, flexibility, sense of control  - VV barriers: restrictive virtual visiting practices, failing expectations, lack of pre-planning, failing to prepare patient, incorrect camera positioning, insufficient staff resources, connectivity, lack of call closure | | |  |  |
| **Author, year (country)** | **Type of Study** | | | **Setting** | | | | **Participants** | | | **Type of intervention** | | | **Outcome** | | | **Results** | |  |
|  |  | | |  | | | |  | | |  | | |  | | | - Recommendations: emotional self-preparation, technology availability | |  |
| Krewulak et al., 2022  (Canada) | Qualitative Study | | | ICU | | | | Patients (*n* = 3)  Family members (*n* = 8)  Nurses (*n* = 17) Physicians (*n* = 13) | | | Telecommunication on ICUs due to visitation restrictions during COVID-19 | | | - Impact of restricted visitation policies  - Strategies facilitating better communication | | | 1) patient and family psychosocial and information needs  2) communication tools  3) quality of communication  4) changing roles and responsibilities  5) facilitators / barriers for alternative communication | |  |
| Otte et al., 2022  (Denmark) | Qualitative Study | | | ICU | | | | Registered Nurses (RN) (*n* = 6) | | | Video calls during COVID-19 lockdown on ICU | | | Nurses’ experiences with video calls | | | - Relatives gain insights into patient treatment without risking infection / having to travel long distances - Patient: “window” into their home, normal everyday life - Nurses: saving time, reducing interruptions in daily routines  - greatest challenge: if failed to use the equipment, ethical aspects related to filming patient | |  |
| Xyrichis et al., 2022  (UK) | Qualitative Study | | | ICU | | | | ICU experienced clinicians (*n* = 17)  Non-ICU-experienced family liaison team members (*n* = 19) | | | Virtual visiting | | | Experiences and perceived benefits of virtual visiting | | | - restore the family unit, facilitate family involvement in care and decision-making, aid in sensemaking  - extend ICU access to geographically distant locations and family members unable to visit in person  - expand FCC in non-pandemic conditions | |  |
| Türkmen & Kebapci, 2022  (Turkey) | Qualitative Study | | | ICU | | | | Physician (*n* = 1)  Nurses (*n* = 8)  Clerk (*n* = 1) | | | Structured Virtual Patient Visit (sVPV) for relatives and patient communication | | | - Experiences of sVPV | | | (1) essential program during the pandemic  (2) contributing to patient's recovery  (3) family-centered care  (4) innovativeness  (5) sustainability  - alleviates patient / family anxiety  - increases patient motivation / healing  - decreases healthcare professionals’ workloads - applicable for all ICU patients: conscious + sedated patients under mechanical ventilation | |  |

| **Author, year (country)** | **Type of Study** | | **Setting** | | **Participants** | | **Type of intervention** | | **Outcome** | | **Results** |
| --- | --- | --- | --- | --- | --- | --- | --- | --- | --- | --- | --- |
| Fritz et al., 2022  (France) | Qualitative Study | | ICU | | Surrogate decision makers (*n* = 26)  Physicians (*n* = 18)  Other family members (*n* = 21)  Other health care providers (*n* = 49) | | Fixed-time daily telephone call from senior physicians to families | | - Perceptions of families and provides regarding digital communication strategy | | - Families: key medical messages understandable, insufficient frequency  - Physicians: functional, reduces emotional load and saves time, absence of task interruptions |
| Bruce et al., 2023  (USA) | Qualitative Study | | Tertiary Care Center | | Family members of inpatients (*n =* 3137) | | Unidirectional digital texting platform to update family members | | - Facilitators - Barriers | | 1) Facilitators: update, connection, continuity of communication, staff compassion / service, reduce phone calls  2) Barriers: feel generic, not needed by regular visitation, perceived delay, inconvenience due to security features |
| Conte et al., 2023  (Sweden) | Qualitative Study | | ICU | | Nurses (*n* = 16) | | - | | intensive care nurses’ experiences of communicating and supporting relatives from a distance while working during visiting restrictions | | - Create order out of chaos: lack of clear routine, adhering to but increasingly questioning visiting restrictions - Providing relatives with Information (Phone conversations with information on the patients’ current health condition): video visits structured approach to guide and support the relatives/patients - Video calls used in combination with audio calls/text messages, but audio calls more common (video calls took longer/more preparation) - nurses felt their support as insufficient: physical/psychological stress - individual and person-centred solutions based a combination of digital and audio tools |
| Jungestrand et al., 2023  (Sweden) | Qualitative Study | | ICU | | Family members (*n* = 21) | | - | | experience of in-person visiting restrictions imposed during the pandemic on family members of patients with COVID-19 | | - ICU staff as bridge: Frequent phone updates appreciated; lack of consistent contact person problematic, information build trust  - Digital aids: Video and audio messages helpful but emotionally complex, remote communication appreciated but seen as insufficient substitute for physical presence |
| **Author, year (country)** | **Type of Study** | | **Setting** | | **Participants** | | **Type of intervention** | | **Outcome** | | **Results** |
|  |  | |  | |  | |  | |  | | - Involvement in care: Some felt excluded; personal items helped maintain connection  - Closeness: Physical separation caused distress even when visits allowed  - Understanding restrictions: Families accepted rules but feared missing critical moments - Suggestions: daily email updates to improve information flow |
| Bradely et al., 2024  (USA) | Qualitative Study | | ICU | | Interprofessional hospital providers (*n* = 48) (nurse, physician, anesthesiologist, internist, surgeon, intensivist) | | - | | * HCP’s perspectives on - caring for ICU patients during the COVID-19 pandemic - the use of virtual care, communication for palliative and end-of-life patients | | - Disruption of roles and responsibilities: attempting to fill the role of the physically absent family members, increased burden through additional phone calls with family - Communication and rapport building: Some believed phones and virtual communication effectively maintained providers’ relationships with patients’ loved ones, but others did not find them sufficient for building trust between providers and family members - Emotional impact of physical isolation on patients: virtual communication not sufficient at the end-of-life care, not always effective at decreasing stress among elderly |
| Cermak et al., 2024  (Canada) | Qualitative Study | | ICU, inpatient/ambulatory services | | Health care providers (*n* = 30) | | - | | HCP’s experiences of how Information and communication technology (ICT) supported changes to clinical care (1) changes to care (2) provisions of care (3) emotions (4) implementation and evaluation (5) lessons learned or for future recommendations | | (1) Supporting in-person communication with patients  (2) Facilitating connection between provider to patient and patient to family: especially at EoL, struggle when lack of physical touch, balance between frequency and staff capacity (3) Providing continuity of care: providing medical updates, efficient and faster communication between the care team and family, increased staff workload, limited experiences for older patients  (4) HCPs highlighted the benefits of digital tools for communication and the need for thoughtful consideration in using ICT |
| **Author, year (country)** | **Type of Study** | | **Setting** | | **Participants** | | **Type of intervention** | | **Outcome** | | **Results** |
|  |  | |  | |  | |  | |  | | - The study emphasizes the importance of education and training for HCPs in using ICT effectively and the need for autonomy in choosing service delivery modalities. |
| Clarke et al., 2024  (UK) | Qualitative Study | | ICU | | Junior doctors (*n* = 8) | | - | | Ethical issues raised by using virtual visiting in the ICU - Fulfilling a moral instinct to connect families, holding hands on behalf of family - Promoting autonomy: nonverbal expression when unwell - Privacy concerns - Managing family’s' emotional distress: greater emphasis on verbal reassurance (challenging, helplessness) - clinical/ethical guidance for virtual visiting: prevent staff from feeling isolated in decision-making | | - Virtual visiting can both improve and worsen ethical aspects of healthcare  delivery in ICUs by fulfilling moral instincts to connect families and promoting autonomy.  - However, it also raises concerns about preserving dignity and privacy, managing emotional distress, and providing equitable access to virtual visiting technologies.  - Recommendations include virtual communication skills training for staff and accessible educational resources for families to address these ethical issues. |
| de Figueiredo et al., 2024  (USA) | Qualitative Study | | Intermediate Care (IMC) | | NA | | Video visitation from family members | | Benefits, barriers, and enhancements of the virtual visitation method | | - benefits: comfort, closure, bridging a gap, reducing loneliness and isolation, bringing home to the hospital, extending the reach of rounding providers - barriers: device challenges, emotional toll on nurses, time intensity, application challenges, technical issues, lack of training, rapid implementation challenges, privacy and security concerns, changes related to the external environment - lack of standard operating procedures |
| Ahmed et al., 2025  (UAE) | Qualitative Study | | ICU | | Family members (*n* = 32) | | - | | - experiences and concerns of family | | - Regularly scheduling audio/video calls: ICU managers should |
| **Author, year (country)** | **Type of Study** | | **Setting** | | **Participants** | | **Type of intervention** | | **Outcome** | | **Results** |
|  |  | |  | | HCPs (*n* =21) | |  | | members and health care professionals facing the challenges during ICU isolation - recommendations for enhancing family support and improving effective communication | | identify alternative options for family members who are unable to use the technology  - Occasional physical presence with patients, such as visits every 2–3 weeks, is preferred by some family members --> HCPs recommend revising the visiting policy to allow family  members to be present in the ICU for extended periods, while considering strict infection control and prevention measures  - Recommend regular family meetings to encourage effective family engagement - Assign someone to address family members' questions --> liaison nurse  - Family members expressed concerns about post-ICU discharge plans, prognosis, and treatment quality, while health care providers recommended expanding the ICU team to include social workers and liaison nurses  - The study emphasizes the importance of family support and involvement in ICU care, suggesting better communication strategies and policy changes to enhance family involvement |
| Blok et al., 2025  (USA) | Qualitative Study | | ICU | | Family caregivers (*n* = 20)  Care team members (*n* = 12) | | - | | - factors and strategies that affect psychological distress and well-being among family caregivers during a critical care hospitalization - Family caregiver: experiences during the ICU hospitalization - Care team member interviews: family engagement, distress during critical care, current practices/opportunities | | - Factors contributing to psychological distress: unfamiliar health care and treatment processes, illness uncertainty, patient appearance, responsibilities related to the caregiver role, unmet expectations around predictable  communication  - Factors supporting caregiver well-being: proactive/personal communication, emotional support, a welcoming and respectful environment - in-person communication, videoconference options as beneficial |
| **Author, year (country)** | **Type of Study** | | **Setting** | | **Participants** | | **Type of intervention** | | **Outcome** | | **Results** |
|  |  | |  | |  | |  | | to provide family support | | and comforting during visitor restrictions  - Differences in perspectives between caregivers and care team members highlight areas for improvement in addressing caregiver needs |
| Johnson et al., 2025  (UK) | Qualitative Study | | ICU | | Former patients (*n* = 5) Family member (*n* = 1)  Nurses (*n* = 3)  Medical staff members (*n* = 3) | | Family Member's Voice Reorientation Intervention (FAMVR) for delirium prevention and management in critically ill adult patients. | | Codesign process | | - Four main content themes: Message content (timely, context-sensitive), message wording (simple, calm, non-threatening), emotional reactions (reduces fear, supports family), message tone (neutral, reassuring about ICU noises and procedures)  - Final intervention includes 4 domains: orientation, personal care, procedures, flexible/personalized messages  - Messages played 3× daily; remote recording possible  - Seen as acceptable, emotionally supportive, and feasible by participants |
| ***Quantitative studies*** |  | |  | |  | |  | |  | |  |
| de Havenon et al., 2015  (USA) | Prospective, nonblinded, nonrandomized pilot study | | ICU | | Family (*n* = 16 Meetings, 88 members) | | Audio-visual technology (Conference calling, Skype) | | Family satisfaction with the meeting + decision making process | | - welcomed in a sizable percentage of family meetings  - no adverse impact on satisfaction or other metrics |
| Gorman et al., 2020  (Australia) | Prospective observational study | | ICU | | Family (*n* = 114) | | Pre-written, real-time SMS-updates at five distinct landmark events | | Feeling of SMS service at 5 timepoints | | - reassuring, informative and easy to follow  - family members would recommend the SMS service  - SMS service didn’t increase anxiety |
| Shahdosti et al., 2020  (Iran) | Randomized parallel group-controlled trial | | ICU | | Coronary artery bypass graft (CABP) patients (*N* = 66, C: 33 / I: 33) | | Digital communication method - Video visitation at least two times per day, for three days | | - Anxiety - Depression symptoms - Hospital Anxiety and Depression Scale (HADS) | | - reduced anxiety  - can be used to reduce postoperative complications  - mental support of patients by their families  - easy to use, cost-effective, decrease referrals to hospitals |
| **Author, year (country)** | | **Type of Study** | **Setting** | **Participants** | | **Type of intervention** | | **Outcome** | | **Results** | |
| Ehrler et al., 2021  (Switzerland) | | Quantitative survey study | ICU | Patients (*n* = 33)  Relatives (*n* = 268)  Caregivers (*n* = 17) | | Videoconference between patient-family | | - Evaluation of satisfaction, intention to use again | | - High satisfaction of patient/family  - mitigate loneliness for patients  - Visio not straightforward, required specific training  - privacy issues → integrated solution: satisfaction, connection | |
| Rose et al., 2021  (UK) | | Multicenter,  cross-sectional  survey study | ICU | Hospitals (*n* = 117) | | Philanthropic COVID-19 tablets project (virtual visiting) | | - family visiting policy during the COVID-19 pandemic - communication strategies for the provision of clinical updates and advanced care planning   communication at the  end of life   - virtual family visiting, including the use of the Life lines virtual visiting solution - communication training + Open-ended comments | | - Benefits of Virtual Visiting (VV):  Promotes patient psychological and physical recovery  Reorients patients with delirium  Overcomes language/communication barriers  Enhances patient-centered care  Improves staff morale  - Barriers to VV:  Family challenges with video-platform technology / accessing suitable devices  - Guidance on Video Communication:  Follow principles of the 2005 Mental Capacity Act  Establish evidence of patient consent for VV  Challenges with documented consent for patients with impaired mental capacity | |
| Erazo-Muñoz et al., 2021  (Columbia) | | Retrospective,  descriptive,  observational study | ICU  Wards | Relatives of suspected COVID-19 patients (*n* = 871) | | Support strategies (virtual visiting, personal visiting) | | - family satisfaction | | - Virtual ward round: second most frequently used modality after personal accompaniment  - Virtual support: Patients' needs for nutrition, specialized information, aids (61.1%) / Family satisfaction: Completely satisfied (35.5%) | |
| Shariati et al., 2021  (Iran) | | Parallel randomized trial | ICU | Family of COVID-19 patients (*N* = 82, I: 41 / C:41) | | Digital communication method (web-based communication) between nurse-family with patient information | | Family member stress:  Perceived Stress Scale (PSS-14) | | - effective in reducing family perceived stress  - safe, inexpensive | |
| Suen et al., 2021  (USA) | | Pilot-RCT | ICU | 52 Patients and their surrogates (*N* = 104, I: 27 / C: 25) | |  | | feasibility, usability, acceptability, perceive  -ed effectiveness of the intervention, quality of communi-  cation and shared decision-making | | - tool was highly usable, acceptable and effective - Compared with the control group, surrogates who used the tool reported higher overall quality of communication, in shared decision-making (not significant) | |
| **Author, year (country)** | | **Type of Study** | **Setting** | **Participants** | | **Type of intervention** | | **Outcome** | | **Results** | |
|  | |  |  |  | | Family support tool 1) surrogates’ completion of Family Support Tool modules before the first two family meetings 2) provision to the ICU team of a one-page summary sheet containing surrogates’ responses to questions presented in the tool 3) scheduled family meetings | |  | |  | |
| Rahul et al., 2022  (Australia) | | Cross-sectional questionnaire study | ICU | Family (*n* = 155)  Physicians (*n* = 205) | | - | | - Family expectations  - Physician perspectives  - Satisfaction | | - more video calls by family than physicians and less deterrent effects of virtual communication to quality of service by family than for physicians  - training sessions on communication skills (particularly virtual means) for physicians recommended | |
| Nelson et al., 2022  (USA) | | Quantitative survey study | ICU | ICU providers (*n* = 176) | | Telemedicine during COVID-19 among providers, families, patients | | - Use of telecommunication on ICU | | - seen as valuable in taking care of patients by 93.8-100.0% of respondents | |
| Kebapci & Türkmen., 2022  (Turkey) | | Observational,  prospective,  single-center study | ICU | Patients (*n* = 50)  Relatives (*n* = 50) | | Structured virtual patient visit (sVPV) program: daily during the stay on ICU (max. 6 months) | | - Patient: Anxiety/depression (VAS-A, Face Anxiety Scale, HADS) - Family: Anxiety/depression (HADS), Satisfaction (Scale for the Assessment of the Satisfaction of the Relatives of the Inpatient Patients (SASRIP)) | | - decreased anxiety in patients under IMV, non-IMV, simple mask/HFNC support  - decreased anxiety of all patients and their relatives  - supported family emotionally | |
| Rose et al., 2022  (UK) | | Multicenter,  prospective, observational cohort study | ICU | Family members who experienced at least one virtual visit during the COVID-19  (*N* = 2166 adult family members of | | Digital communication intervention (second COVID-pandemic wave) | | - Family distress (Distress thermometer) - Depression, anxiety and stress (Depression, | | - VV reduced distress  - most common emotions reported after VV: feeling reassured / relaxed  - least common emotions: anger / fear | |
| **Author, year (country)** | | **Type of Study** | **Setting** | **Participants** | | **Type of intervention** | | **Outcome** | | **Results** | |
|  | |  |  | ICU patients from 37 hospitals) | |  | | Anxiety and Stress Scale (DASS-21))   - Discrete Emotions Questionnaire (DEQ) | |  | |
| Sanfilippo et al., 2022  (Italy) | | Prospective pilot study | ICU | Family (*n* = 20) | | OSIRIDE (Video-calls between patients & family): family members in turn can send photos, music, videos | | - Family anxiety, depression (HADS), Post-traumatic stress disorder (PTSD) (Impact of Event Scale (IES-R)) | | - no significant difference in depression, anxiety, PTSD  - Video calls between family-patients in ICUs are feasible | |
| Shirvani et al., 2022  (Iran) | | Quasi experimental study | ICU | Family (*n* = 60, C: 30 / I: 30)  Patient (*n* = 60, C: 30 / I: 30) | | Telehealth communication (5 days): reporting patient’s condition by audio / video messages to family | | - Family depression,  anxiety (HADS) | | - Patient status communication reduces severity of family anxiety / depression  - liaison psychiatrist communication reduced face-to-face visits | |
| Uysal et al., 2023  (Turkey) | | Quasi experimental study | ICU | Patients (*n* = 135)  Relatives (*n* = 135) | | Video Call (patient-family)  - 5 days/a 10min in average | | - Vital signs  - Video call satisfaction | | - increase on HR, RR, and GCS  - effect on PR  - no effect on BP, SpO2  - Patients-relatives very satisfied | |
| Jeitziner et al., 2023  (Austria, Germany, Switzerland, Liechtenstein) | | Quantitative descriptive study | ICU | *N* = 151 (patients, family, HCP) | | - | | - opinions and experiences on challenges in family-centred care for intensive care unit patients and affected families during the pandemic | | - High agreement on: Daily structured updates, respectful communication, dedicated contact person, use of plain language, staff communication training  - Low agreement on: Pre-pandemic video calls, sending photos of ventilated patients, text chats with families  - Challenges noted: time/resource burden for staff, family's limited technical skills, emotional strain from video/images  - Recommendations: ICU diaries, structured communication tools (e.g. checklists), clear protocols for virtual contact (secure platforms, staff roles), Web-based info resources for families (e.g. ICU walkthroughs, hygiene, delirium), consider post-ICU family support needs, sustain helpful innovations post-pandemic | |
| Levido et al., 2023  (Australia) | | Quantitative descriptive study | ICU | HCP (*n* = 106)  Family member (*n* = 69) | | Virtual meeting/visitation on ICUs | | - reported experiences - satisfaction surrounding the use of | | - Health Care Workers: mostly audio-visual calls; main purpose: virtual visits (79%), Perceived anxiety reduction: | |
| **Author, year (country)** | | **Type of Study** | **Setting** | **Participants** | | **Type of intervention** | | **Outcome** | | **Results** | |
|  | |  |  |  | |  | | technology for virtual visits and virtual family meeting | | mean 6.9/10, Non-verbal cue recognition: mean 6.8/10  - Family Members (n = 69): Communication for family meetings (41%) or updates (36%), Perceived benefit: mean 9.4/10, 95–98% supported repeat and continued use, felt included and able to ask questions | |
| Yuan et al., 2023  (China) | | Randomized controlled trial | ICU | Patients (*N* = 121, I: 65 / C: 56)  Family (*N* = 98, I:50 / C: 48) | | Video visitation (every afternoon): WeChat | | - Patients: Anxiety/depression symptoms (HADS), Delirium incidence  - Family: Anxiety/depression symptoms (HADS), Satisfaction | | - no reduction of patient anxiety, depression, incidence of delirium  - improved satisfaction (patient, family) | |
| Pozzoli et al., 2023  (Switzerland) | | Randomized controlled trial | Cardiac surgery ward | Patients (*N* = 48, (I: 32, C: 16)) | | communication software for families to receive daily interprofessional (medical, nursing, and physiotherapy) updates by text message on patients’ postoperative clinical state | | - Satisfaction with communication - Outgoing vs. incoming communications (Call back rate, SMS) | | - Satisfaction with communication: Digital: 6.7 vs. Standard: 5.6 (p = 0.004)  - Lower call-back rate in digital group (34% vs. 75%), Mean 4.84 SMS/patient sent  - SMS especially appreciated during early postop days (Day 2–3)  * Digital updates increased satisfaction and reduced family-initiated calls  High acceptance; feasible implementation  No interference with private communication by mobile phones SMS perceived as clear, informative, and emotionally reassuring | |
| Ramirez et al., 2024  (USA) | | Quantitative descriptive study | ICU | Family members (*n* = 82) | | Virtual family centered rounds (FCR) with tablets or phone calls | | Family members’ perceptions of the benefits and barriers of virtual FCRs. | | - Most family members valued the treatment explanations provided and invitation to participate in care  - More than 75% of respondents perceived the highest level of care in trust, communication, relationship, and compassion with their provider. - A minority of family members felt overwhelmed with medical language used during virtual FCR | |
| **Author, year (country)** | | **Type of Study** | **Setting** | **Participants** | | **Type of intervention** | | **Outcome** | | **Results** | |
| Fernández et al., 2024  (Spain) | | Quantitative descriptive study | ICU | ICUs (*n* = 52) | | - | | - modifications during SARS-CoV-2 pandemic to the policy of visits to ICU patients in general, specifically in end-of-life accompaniment - the use of new communication technologies between relatives, patients and professionals | | - use of new technologies increased significantly with the pandemic (59.6% --> 96.2%) - mobile phones of relatives/patients were preferentially used in the pre-pandemic state, 20% of the time during the pandemic other devices exclusively for this purpose, or combination of both - use of virtual contacts between patients and relatives increased significantly compared to the pre-pandemic | |
| Woo et al., 2024  (South Korea) | | Randomized controlled trial | ICU | Patients and family member couples (*N* = 38, I: 18, C: 20) | | Daily 15-minute virtual visits (Zoom), up to 7 days | | - Family satisfaction (FS-ICU 24)  - Patient anxiety and depression (HADS) | | - Family satisfaction significantly higher in virtual visit group (FS-ICU 24: 89.1 vs. 75.1; p = 0.03)  - Greater reductions in HADS-Anxiety (–59.4% vs. –15.4%) and HADS-Depression (–64.5% vs. –24.2%), both p < 0.001  - Conclusion: Virtual visitation improves family satisfaction and reduces psychological distress in ICU patients during pandemic restrictions | |
| Shin et al., 2025  (USA) | | Quantitative pilot study | ICU | Family care giver (*N* = 28, I:14 / C:14) | | VidaTalk™: touchpad tablet computer software app developed to help MV patients communicate feelings, needs, and questions to care providers and families (voice output, common needs/requests, feelings/symptoms, questions, pain messages (location, intensity rating, and quality), a keyboard, a finger drawing pad) | | - HADS (Hospital Anxiety and Depression Scale) - PTSD-related symptoms (Impact of Event Scale-revised - Family perceived communication difficulty (Family Communication Scale (FCS)) | | - VidaTalk may be associated with a lower level of anxiety and PTSD-related symptoms in ICU family care givers post-ICU discharge | |
| Murray et al., 2025  (Ireland) | | Quantitative descriptive study | ICU | Nursing staff (*n* = 22) (primary nurses, facilitators of the system) | | Ad hoc web-based bedside visits available 24/7  with 1 or more close family members (“ICU Family-  Link”) | | - modified Telehealth Usability questionnaire (mTUQ): ease of use, learnability, interface quality, interaction quality, reliability, satisfaction, future use, usefulness - Participants use | | - Overall positive responds to all subcategories - Organizing the web-based visit could be extra staff workload - good interface quality - good interaction quality, also dependent on family network coverage - very useful, satisfied, willing to use in the future | |
| **Author, year (country)** | | **Type of Study** | **Setting** | **Participants** | | **Type of intervention** | | **Outcome** | | **Results** | |
| Bendavid et al., 2025  (Israel) | | Randomized controlled trial | ICU | Patients (*N* = 60 (I: 30 / C: 30)) | | EyecontrolMed: up to 7 days  of device usage, 8 hours daily, receiving orientation messages, family messages, music/religious contents according to their preference and white noise. | | Delirium severity (CAM-ICU-7) | | - positive trend for decreased delirium in ventilated patients - significant improvement in delirium for patients above 50 | |
| ***Mixed-methods studies*** | |  |  |  | |  | |  | |  | |
| Hoffmann et al., 2019  (Austria) | | Mixed-methods study | ICU | Laypersons (*n* = 20)  Experts (*n* = 10) | | Dynamic online information platform website for ICU patient relatives (ICU facts, communication advice, FAQs, family diary, children’s section, videos, support resources) | | - usability/functionality of the ICU family’s website | | - High usability ratings: navigation, clarity, relevance of content (mean scores ~8.4–9.1/10)  - Participants valued the diary, inclusion of children, and user-friendly structure  - Criticism: lack of images, no home button, some links too hidden  - Professionals would recommend the site to relatives (mean 9.1/10)  - Suggestions: clearer icons, stronger visual elements, easier navigation | |
| Zante et al., 2022  (Switzerland) | | Prospective single-center mixed-methods study | ICU | Relatives (*N* = 52 (1:1=I:C)) | | Virtual communication method (Video call frequency agreed individually with each relative) | | - PTSD symptoms (IES-R)  - Family satisfaction (adapted Family Satisfaction in ICU 24-Item-Revised questionnaire (aFS-ICU 24R)) | | - no significant effect on PTSD symptoms in relatives  - Family satisfaction no difference | |
| Bansal et al., 2022  (Canada) | | Mixed-methods program evaluation study | Tertiary care center | - initial needs assessment survey: healthcare professionals (*n* = 139)  - after‐use survey: any users of the devices (*n* = 31)  - Exit survey: healthcare staff employed regardless of whether they had used devices to better understand cases and barriers (*n* = 47) | | Virtual technology program: communication between patients, families and care team | | - Comparison anticipated needs vs. actual use  - Frequency of use across sites  - User experience / satisfaction  - Most common reasons for using the device  - Strengths / areas of improvement | | - useful program  - gap in ease of use  - potential facilitator: nursing /social work staff in device use | |
| **Author, year (country)** | | **Type of Study** | **Setting** | **Participants** | | **Type of intervention** | | **Outcome** | | **Results** | |
| Aledreesi et al., 2023  (SAU) | | Mixed-methods cross-sectional study | ICU | Family (*n* = 54) | | Telehealth communication app (myVisit) | | - Family: Usability (System Usability Scale (SUS)), satisfaction | | - System usability very good (71%)  - users' opinions save time (96%), save money/effort for family (74%)  - limited time for use | |
| Rosenthal et al., 2024  (UK) | | Mixed-methods study | Inpatient settings including ICU | Patients (*n* =33)  Family (*n* = 145)  Providers (*n* = 36) | | “Inpatient Telehealth Program” to virtually bring care team providers and family members to the bedside | | - Implementation assessment of the Inpatient Telehealth Program from the perspectives of patients, families, care team providers | | * Inpatient telehealth - enhanced the patient and family experience through strengthened relationships and increased support: family members’ wellbeing, addressed unique needs - low awareness of the program limited adoption of inpatient telehealth: impeded workflow - Could be provided to all admissions - Integrating it into conversations around hospital visitation policies, need for additional program resources for equitable access by all families, including those lacking broadband and smart devices, clerical support for the program to promote expansion and minimize nurses' burden | |
| Johnson et al., 2025  (Australia) | | Mixed-methods pilot study | ICU | Patients (*n* = 15)  Family (*n* = 15)  - qualitative component: *n* = 17 (three patients, six family members, eight nurses) | | Family member's voice reorientation (FAMVR) intervention that digitally records familiar voices to provide auditory orientation to mechanically ventilated critically ill adult patients | | - Richmond Agitation-Sedation Scale (RASS) - Experiences of patients, family members, clinical staff (feasibility, acceptability of FAMVR) | | - no negative impact on RASS - Participants found the FAMVR intervention feasible and valuable - facilitated good communication - increased knowledge of delirium by nursing staff - recording messages emotionally challenging, but ultimately perceived as beneficial, offering reassurance, comfort - improve patients' alertness, cognitive function - supported person-centred care - nurses challenges in documentation on electronic medical records (EMR) - Patients generally returned to baseline well-being post-intervention - family members/ nurses psychologically not affected | |
| Atasoy et al., 2025  (Turkey) | | Mixed-methods study | ICU | Patients (*N* = 93 (I: 47/ C: 49)) | | Interaction model (delivery of videos, images and | | - Standardized Mini Mental Test | | - anxiety levels of patients in the intervention group exhibited a decline | |
| **Author, year (country)** | | **Type of Study** | **Setting** | **Participants** | | **Type of intervention** | | **Outcome** | | **Results** | |
|  | |  |  |  | | messages conveyed by the patients' relatives to the patients and subsequent transfer of these patients' messages to their relatives) | | (cognition) - Four Score (Full Outline of Unresponsiveness  Score) (coma) - Beck Anxiety Inventory (BAI) - Warwick-Edinburgh Mental Well-Being Scale | | over time, whereas those in the control group demonstrated an increase - mental well-being of patients in the intervention group exhibited an upward trajectory over time, whereas the control group demonstrated a decline in this metric - fear, uncertainty (anxiety), hopelessness, positive emotions after intervention | |

*Extracted data included author(s), year, country, study design, population, type of telecommunication, main outcomes, and key findings. Data extraction was conducted independently by two reviewers (E.O., N.G.) following JBI guidelines for mixed-methods systematic reviews.*

**Abbreviations**: HCP - health care professional; ICU - intensive care unit; VV - virtual visiting; sVPV - structured virtual patient visiting; FST - family support team; RCT - randomized controlled trial; QoL - quality of life; PCC - patient-centred care; PFCC - patient- and family-centred care; PTSD - post-traumatic stress disorder; HR - heart rate; RR - respiratory rate; GCS - Glasgow Coma Scale; PR -pulse rate; SD - standard deviation; SMD - standardized mean difference; MD - mean difference; CI - confidence interval.

**Table S2**. Critical appraisal results of eligible studies using Mixed-Methods Appraisal Tool (MMAT).

| **Author, year** | **Critical appraisal of eligible qualitative studies** | | | | | | | |
| --- | --- | --- | --- | --- | --- | --- | --- | --- |
|  | Q1. Is the qualitative approach appropriate to answer the research question? | | Q2. Are the qualitative data collection methods adequate to address the research question? | | Q3. Are the findings adequately derived from the data? | Q4. Is the interpretation of results sufficiently substantiated by data? | Q5. Is there coherence between qualitative data sources, collection, analysis and interpretation? | |
| Dhala et al., 2020 | Y | | CT | | Y | Y | | Y |
| Dhahri et al., 2021 | Y | | Y | | Y | Y | | Y |
| Kennedy et al., 2021 | Y | | Y | | Y | Y | | Y |
| Sasangohar et al., 2021 | Y | | Y | | Y | Y | | Y |
| Klop et al., 2021 | Y | | Y | | Y | Y | | Y |
| White et al., 2021 | Y | | N | | Y | Y | | Y |
| Rose et al., 2022 | Y | | Y | | Y | Y | | Y |
| Krewulak et al., 2022 | Y | | Y | | Y | Y | | Y |
| Otte et al., 2022 | Y | | Y | | Y | Y | | Y |
| Xyrichis et al., 2022 | Y | | Y | | Y | Y | | Y |
| Türkmen & Kebapci, 2022 | Y | | Y | | Y | Y | | Y |
| Fritz et al., 2022 | Y | | Y | | Y | Y | | Y |
| Bruce et al., 2023 | Y | | Y | | Y | Y | | Y |
| Jungestrand et al., 2023 | Y | | Y | | Y | Y | | Y |
| Conte et al., 2023 | Y | | Y | | Y | Y | | Y |
| Bradley et al., 2024 | Y | | Y | | Y | Y | | Y |
| Clarke et al., 2024 | Y | | Y | | Y | Y | | Y |
| Cermak et al., 2024 | Y | | Y | | Y | Y | | Y |
| de Figueiredo et al., 2024 | Y | | Y | | CT | CT | | CT |
| Ahmed et al., 2025 | Y | | Y | | Y | Y | | Y |
| Blok et al., 2025 | Y | | Y | | Y | Y | | Y |
| Johnson et al., 2025 | Y | | Y | | Y | Y | | Y |
|  |  | | | | | | | |
| **Author, year** | **Critical appraisal of eligible quantitative studies** | | | | | | | |
| **Quantitative descriptive studies** | Q1. Is the sampling strategy relevant to address the research question? | | Q2. Is the sample representative of the target population? | | Q3. Are the measurements appropriate? | Q4. Is the risk of nonresponse bias low? | | Q5. Is the statistical analysis appropriate to answer the research question? |
| Rose et al., 2021 | Y | | Y | | Y | Y | | Y |
| Rahul et al., 2022 | Y | | Y | | Y | CT | | Y |
| Nelson et al., 2022 | Y | | Y | | Y | Y | | Y |
| Levido et al., 2023 | Y | | N | | Y | N | | Y |
| Jeitnizer et al., 2023 | Y | | CT | | Y | CT | | Y |
| Fernandez et al., 2024 | Y | | Y | | Y | N | | Y |
| Ramirez et al., 2024 | Y | | N | | Y | N | | Y |
| Murray et al., 2025 | Y | | N | | Y | N | | Y |
| **Quantitative non-randomized studies** | Q1. Are the participants representative of the target population? | | Q2. Are measurements appropriate regarding both the outcome and intervention (or exposure)? | | Q3. Are there complete outcome data? * | Q4. Are the confounders accounted for in the design and analysis? | | Q5. During the study period, is the intervention administered (or exposure occurred) as intended? |
| de Havenon et al., 2015 | N | | Y | | Y | N | | Y |
| Gorman et al., 2020 | Y | | Y | | N | CT | | Y |
| Ehrler et al., 2021 | CT | | Y | | Y | CT | | Y |
| Erazo-Muñoz et al., 2021 | Y | | Y | | Y | CT | | Y |
| Kepabci & Türkmen, 2022 | Y | | Y | | Y | N | | Y |
| Rose et al., 2022 | Y | | Y | | Y | N | | Y |
| Sanfilippo et al., 2022 | N | | Y | | Y | N | | Y |
| Shirvani et al., 2022 | Y | | Y | | Y | Y | | Y |
| Uysal et al., 2023 | Y | | Y | | Y | N | | Y |
| Shin et al., 2025 | N | | Y | | Y | N | | Y |
| **Quantitative randomized controlled trials** | Q1. Is randomization appropriately performed? | Q2. Are the groups comparable at baseline? | | Q3. Are there complete outcome data? | | Q4. Are outcome assessors blinded to the intervention provided? | | Q5. Did the participants adhere to the assigned intervention? |
| Shahdosti et al., 2020 | Y | Y | | Y | | CT | | Y |
| Shariati et al., 2021 | Y | Y | | Y | | CT | | Y |
| Suen et al., 2021 | Y | Y | | Y | | CT | | Y |
| Yuan et al., 2023 | Y | Y | | Y | | Y | | Y |
| Pozzoli et al., 2023 | Y | Y | | Y | | Y | | Y |
| Woo et al., 2024 | Y | Y | | Y | | CT | | Y |
| Bendavid et al., 2025 | CT | CT | | Y | | CT | | CT |
|  |  | | | | | | | |
| **Author, year** | **Critical appraisal of eligible mixed-methods studies** | | | | | | | |
|  | Q1. Is there an adequate rationale for using a mixed methods design to address the research question? | | Q2. Are the different components of the study effectively integrated to answer the research question? | | Q3. Are the outputs of the integration of qualitative and quantitative components adequately interpreted? | Q4. Are divergences and inconsistencies between quantitative and qualitative results adequately addressed? | Q5. Do the different components of the study adhere to the quality criteria of each tradition of the methods involved? | |
| Hoffmann et al., 2019 | Y | | Y | | Y | CT | | N |
| Zante et al., 2022 | Y | | N | | N | N | | N |
| Bansal et al., 2022 | Y | | Y | | Y | Y | | Y |
| Aledreesi et al., 2023 | Y | | Y | | Y | Y | | Y |
| Johnson et al., 2025 | Y | | Y | | Y | Y | | Y |
| Rosenthal et al., 2025 | Y | | Y | | Y | Y | | Y |
| Atasoy et al., 2025 | Y | | Y | | Y | Y | | Y |

*Methodological quality was assessed independently by two reviewers (E.O., N.G.) using the Mixed Methods Appraisal Tool (MMAT, version 2018). Each study was evaluated according to five design-specific criteria. Following MMAT guidance, studies were not scored numerically but categorized descriptively as high, moderate, or low quality for narrative purposes.*

**Abbreviations**: MMAT - Mixed Methods Appraisal Tool; RCT - randomized controlled trial; NR - not reported; NA - not applicable; Y - yes (criteria met); N - no (criteria not met); CT - can’t tell (unclear or insufficient information).

* Complete outcome date refers to 80% of the participants and less than 20% dropout rates as acceptable for follow-up measurements.
